# Analyzing group communication dynamics and content in a common-pool resource experiment

**DOI:** 10.1371/journal.pone.0283196

**Published:** 2023-05-02

**Authors:** Patrick Hoffmann, Sergio Villamayor-Tomas, Maria Claudia Lopez

**Affiliations:** 1 Institut de Ciència i Tecnologia Ambientals (ICTA), Universitat Autònoma de Barcelona, Cerdanyola del Vallès, Spain; 2 Department of Community Sustainability, Michigan State University, East Lansing, MI, United States of America; Universidad de Murcia, SPAIN

## Abstract

We study costly communication in a common-pool resource (CPR) experiment as a proxy for two different forms of participatory processes: as a public good and as a club good. A public communication meeting, representing centralized participatory processes, occurs when all group members’ monetary contributions reach a specified threshold. Club communication meetings, representing networked participatory processes, follow only among those members of the group who pay a communication fee. We test whether the way costly communication is provided affects the willingness of participants to contribute to communication, as well as the dynamics of such payments, and the content of communication. This is done by analyzing contributions to communication and communication content of 100 real-life resource users participating in a lab-in-field experiment. We find that contributions towards communication are higher when communication is public, and that club communication features more frequent but less inclusive communication meetings. Also, communication content is more oriented towards addressing the collective action problem associated with the management of the resource when communication groups are attended by all participants. The identified differences between the two ways to provide for communication can inform policies and the design of participatory processes in natural resource governance.

## Introduction

In the context of the current climate crisis and an ecological emergency [1, 2], it is urgent to better understand the behavior of users of natural common-pool resources (CPRs) and contribute to the design of better institutions to alleviate the social dilemmas they confront [3]. A large number of laboratory and lab-in-field economic experiments have been conducted to assess human behavior in dilemma situations, both in the provision of public goods [4, 5] and in the appropriation of CPRs [6]. Similarly, a large body of literature has addressed the role of institutions in alleviating social dilemmas, including social norms and rules [7, 8]. In addition, a vast amount of evidence suggests that introducing the possibility for individuals to communicate in public good and CPR situations enhances cooperation [9–11]. Providing a communication platform to improve participatory processes is an attractive policy alternative, as it does not entail the degree of social opposition or administrative costs that regulation or taxes can generate; however, it requires an investment in time and money for participants, and the decision-making process itself can be challenging to achieve [12–15].

In this study, we explore the behavioral implications of alternative institutional set-ups to provide for costly communication. In our *public communication* set-up, we conceptualize communication as a public good, i.e., as subject to non-excludability and non-rivalry once it is provided for. Thus, public communication is guaranteed once the group reaches a monetary contribution threshold regardless of the individual contributions to communicate. In our *club communication* set-up, we conceive communication as a club good, i.e., as subject to excludability but non-rivalry (i.e., within the club). Those who contribute to the provision of communication benefit from it by communicating with each other, whereas those who do not pay for communication are excluded. Both formats can be understood as proxies for a centralized and networked participatory process, respectively, as will be detailed further below.

Many authors have explored the impact of free communication in public good or CPR games [16–18]. In reality, however, individuals face temporal, spatial, and financial restrictions that can prevent communication from arising. Engaging in communication takes time, may require travelling, or involve organizational costs [19], which motivated scholars to study the implications of costly communication in dilemma situations. In experimental research, costly communication was first introduced in the form of a nested public goods problem [20], i.e., on top of a public good dilemma, individuals faced the problem of enabling communication through voluntary contributions from their endowment. Other authors such as [21], designed communication in a resource appropriation game such that players had to give up harvesting time to communicate, hence representing an opportunity cost. Alternatively, in a minimum-effort coordination game, [22] found that imposing a small fee on communication reduces its emergence. A partial subsidy to cover the fee, however, partly reversed this effect.

Communication is often characterized as a public good [23]. This is the case, for example, when a public or private agency/organization organizes an open communication process to enhance participation among stakeholders, i.e., a centralized participatory process [24, 25]. However, communication can also adopt the characteristics of a club good. In many occasions, communication among stakeholders occurs as a byproduct of social networks, i.e., only those who know each other and who are willing to share information or intentions will end up communicating [26–29]. Or, and particularly the case in large systems, stakeholders attend informal meetings through a networked participatory process, not knowing in advance who and how many will also attend. To our knowledge, there are no studies modeling communication as a club good or testing the differences between public and club communication. More importantly, there is little understanding of whether different ways to provide for costly communication have implications on the emergence and experience of communication, and therefore on the design of communication platforms and policies. We address this gap by analyzing data from a CPR lab-in-field experiment conducted with 100 local water users in the Coello watershed in rural Colombia. The Coello watershed is famous for its agriculture and livestock as the region produces 30% of the fruits and vegetables of the country, and the lower part of the watershed produces rice that requires a lot of water [30]. By the time of the experiments, the majority of the participants said the watershed did not have any water quantity problems. However, due to human-driven activities (i.e., mining activities and deforestation) in the upstream parts of the watershed [31], and the need for irrigation in the lower part (to produce rice and cotton) [30], there is growing concern about future scarcity problems. Different organizations, including the NGOs *WWF Colombia* and *Semillas de Agua*, and the environmental authority of the department *Cortolima* (Corporación Autónoma Regional del Tolima) have worked with local dwellers in rural and urban areas of the watershed to promote better management of the water resources (for details on the study site and background see [32]). The participants in these experiments were rural dwellers from different streams of the watershed, and these experiments were part of a series of activities that this group of organizations were doing to facilitate communication between the different stakeholders present in the region regarding water management and ways to avoid problems in the future. Once all the experiments in the watershed were done, the results were used in a workshop aiming to discuss the importance of collective action in the watershed.

In our lab-in-field experiment we model the *public communication* treatment as a threshold public good whereby everybody is allowed to communicate with each other if a threshold of contributions is reached, regardless of who has contributed and by how much. In the *club communication* treatment, the possibility to communicate is allowed only to those who have paid an individual fixed fee. To assess whether there are differences across the two treatments in terms of the emergence of communication, we analyze monetary contributions to communication meetings. To assess differences in terms of the communication experience, we systematically analyze the content of communication.

We find that the amount of contributions to reach communication is generally higher when communication is public. Furthermore, we observe that the frequency of communication meetings and the size of communication groups vary between both treatments. Club meetings emerge more often but never reach full group size. The analysis of the communication content shows significant differences, with *public communication* groups having more talks addressing the dilemma situation. Finally, the dynamics of meetings over time also differ across both treatments.

## Methodology

### The lab-in-field experiment

The participants were all real-life CPR users familiar with the problematic of watershed management in their communities. The experiment has a linear incentive CPR design, following [32]. According to the formal setting, a group of *n* players make extraction decisions in a CPR, a hypothetical watershed of size *R*_*j*_. Every individual *i* in the group is endowed with an amount of *e* experimental currency units (ECUs), which can be invested in the extraction of the CPR, or in another activity that does not affect the CPR. Each unit invested in the extraction of the CPR, *z*_*i*_, where *z*_*i*_∈(0, *e*), yields to an individual return of *w* ECUs and reduces the CPR size in *c* ECUs. The other activity not affecting the CPR yields a return of *α* ECUs without imposing the negative externality to others in the group that the extraction of the CPR generates. At the end of a round, the aggregate extraction of all individuals in the group is $z_{i}=\sum_{1}^{n}z_{i}$. Also, all remaining units of the resource in the CPR $\left(R_{j}-c\sum_{1}^{n}z_{i}\right)$ are evenly distributed among the *n* players. Thus, the payoff for each player *i* playing with an initial resource size *j* in a round is shown in Eq 1.


$$\pi_{ij}(z_{i})=wz_{i}+\alpha (e-z_{i})+\frac{R_{j}-c\sum_{1}^{n}z_{i}}{n}$$ (1)

The extraction decision becomes a social dilemma if we assume $\left(w-\frac{c}{n})>\alpha >(w-c\right)$. That is to say that the marginal benefit from investing in the activity not affecting the CPR $w-\frac{c}{n}>\alpha$ is lower than the marginal benefit from investing in extraction. The dominant strategy for any individual *i*, the Nash equilibrium, is to invest exclusively in CPR extraction, *z*_*i*_ = *e*, while the social optimum results from all players extracting 0 from the CPR.

The initial individual endowment for each individual was *e* = 20 tokens. Each unit extracted from the CPR *w* had a marginal value of 2 ECUs and reduced the value of the CPR by *c* = 3 ECUs. Each unit invested in the activity not affecting the CPR *α* had a private value of 1 ECU. The size of the CPR, *R*_*j*_ was 675 for the first 10 rounds.

The experiment included two sets of rounds. The first 10 rounds (rounds 1–10) were played without the possibility for the players to communicate, whereas the second 10 rounds (rounds 11–20) allowed for communication at a cost. We had two types of treatments to investigate costly communication. One *public communication* (PC hereafter) treatment, with communication structured as a public good, and a *club communication* (CC hereafter) treatment, with communication structured as a club good. The PC treatment allowed for communication if the group reached a monetary threshold that allowed the group to communicate. The target was set to be *Y*, and individuals could contribute to this target from their own payoffs (collected in rounds 1–10) in that particular round in any amount *y*_*i*_ *ϵ* (0, *Y*). Once the target was met, all individuals in the group were allowed to participate in the discussion for that round regardless of whether they had contributed to communication or not. If the group exceeded the target (the group could meet), or if they did not reach the target (the group could not meet), those contributions were not reimbursed. Like in many threshold-public good situations [33, 34] we wanted to allow for the possibility that some participants contribute more than others. This aimed at recreating communication situations where some individuals or organizations lead participatory decision-making processes.

The CC treatment allowed only those participants who paid a fixed communication fee *v* to communicate. Communication in this treatment was possible if at least two players invested the required amount, but these were the only players that could communicate. If only one player paid for the right to communicate their contributions were not reimbursed. Fixed fees also align with the logic of club goods in real life, as e.g., charging fixed fees to participate in conferences or equally sharing the costs of communication networks as they emerge.

For the second sets of rounds of experiments the size of the resource *R*_*j*_ was 450 (The decreasing resource size was another feature of the experiment and is analyzed in [32]. For this study, we believe that this feature does not affect our analyses of contributions to and content of communication since the two communication treatments remain comparable). The experimental design was such that to achieve the target *Y* in the PC treatment in an equitable way, all individuals had to contribute the fixed communication fee *v* of the CC treatment. In other words, the most proportional way to enable full group communication for both treatments was for each player to invest *v* ECUs. In both cases, providing the communication possibility originated another social dilemma. However, the possibility to communicate does not alter neither the Nash equilibrium, nor the social optimum regarding extraction behavior. In our experiment we set *Y* = 100 and *v* = 20. In both treatments, if communication was allowed, participants discussed for three minutes, and the conversations were recorded with their authorization.

### Experimental procedures

A total of 100 local water users participated in the experiment, which were split in groups of five individuals. In sum we ran the experiment with 20 groups, 10 groups for each treatment. The recruitment process was done by word of mouth with the help of local leaders and *WWF Colombia* and *Semillas de Agua*, that invited water users older than 18 years old to participate in a “decision-making activity”. Once in the experiments, we did not allow people from the same household to participate in the same group. To facilitate participation, transportation to the site where the experiment took place was offered. The experiments were conducted with paper and pencil. As mentioned above, this watershed was not facing a water problem when we ran the experiments, but due to anthropogenic reasons such as mining, deforestation and irrigation, environmental authorities and NGOs were concerned with possible supply problems in the future. In fact, as reported in Table 1, 82% of them have been invited to meetings to discuss water management with their neighbors. We believe this is a strength of our site selection because it allows us to assume certain homogeneity among participants (73% of them have lived in the region more than 10 years) with regards to water variability, and therefore the experiments capture how they will behave and the role that our two communication processes could have if they have to face scarcity in real life. This increases the external validity of the results, possibly expanding their applicability to many other communities that have not experienced water scarcity so far, but might do so in the future.

**Table 1:** Subjects’ socioeconomic characteristics.

Number of participants	100 subjects
Percent male	41%
Mean age	34 years
Percentage of people living in the area for over 10 years	73%
Mean years of formal education	9.4 years
Percentage of people who work on an activity related to natural resources extraction	37%
How often do you discuss with your neighbors’ problems related to water management?	82%

In each session, we ran the experiment with up to three groups of five subjects living in the same community. As is habitual in social dilemma experiments conducted in the field, subjects could identify the other participants in their group but could not observe their decisions or discuss with them other than when communication was allowed [35, 36]. Socio-demographic characteristics of the subjects are presented in Table 1.

Before the start of the instructions, we provided some details about our project and the reasoning to include a payment for the decisions made during the experiment. The conversion of the final payment from ECUs to Colombian Pesos was 1:4. We then followed to read the consent form. We read the instructions aloud (see S1 File), and we asked participants to raise their hands if they had any questions. All questions were answered in public. During the instructions we informed participants about the duration of the experiment, and we provided a detailed description of the decisions making process they were going to make (i.e., extracting resources from a shared watershed or in another option not affecting the watershed), and their payoff function. Due to the static nature of our design, we explained that extractions in one round did not affect the size of the resource in the next round. After the instructions, participants were informed that they could leave the experiment any time during the session. After that, participants played several practice-rounds to become familiar with the game and the various forms. Then the experiment began.

In each round, subjects had to write down their decision on a “harvest decision card”. Then we collected the decision cards, calculated the total extraction from the CPR per group and announced it to all players in a group, along with the final size of the resource for that round, and the resource share earned by each participant in the group. Then each participant had to calculate his or her payoff in that round. For participants with difficulties either writing or doing the calculations, we had assistants helping them writing their decisions or calculating their earnings. During the first ten rounds of the experiment, participants were not allowed to communicate in any way.

In rounds 11–20, we implemented the communication treatments. If more than one group was in the room during a session, all groups within a session were exposed to either the public or the club communication treatment. Whenever a group or a set of participants provided themselves with the right to communicate, they moved to separate rooms so nobody else could hear them. The conversations were audio recorded after requesting verbal consent from the participants (which was also recorded). In the PC treatment, participants got together to communicate, but they could not know who paid for them to communicate (or who did not pay), and in the CC treatment, participants could see who communicated with whom (i.e., who had paid for communication). In both treatments, participants were allowed to discuss their past decisions and to create strategies for future decisions, but they were not allowed to show their previous decisions forms. During these treatments, we added a second “communication decision card” for participants to write down their communication investments. In each round during the treatments, participants were first asked to make their communication decision. Then we calculated and announced the total investments in communication per group, so the groups/subsets of participants entitled to communicate had their chance to do so. After the communication interaction, the round continued normally with the harvest decisions.

### Content analysis of communication interactions

Although analyzing communication content in small group research has a long and rich history [37, 38], doing so in resource dilemmas in the field is only nascent [39]. Early works developed rather rudimentary (although groundbreaking) approaches to content analysis [18]. More recent works have notably expanded the number and diversity of analytical categories, including the distinction of topical and functional category groups [40], proposals, opinions, references and appeals [41], the degree of verbal punishment [42], or the target of talks [43]. In this paper we adopt the distinction of topic and function categories, and follow more specifically those developed in [11].

In order to content analyze the communication events, we transcribed all the communication audio-records. During the transcription process it was possible to assign statements to individuals, but not to identify the individuals personally, or to link statements with their behavior and payoffs in the game. The coding was conducted collaboratively by the first two authors for a first sample, and after agreement on the coding technique, the main part of the coding was conducted by the first author with random checks from the second author. First, the statements of participants were subdivided into coding units identified after the criteria specified in [11, 44]. Specifically, the ensemble of communication transcripts from each experimental group constituted our context units; within each context unit, the pieces of text referring to each of the discussions in a round constituted our sampling units; and the statements within each sampling unit constituted our coding units, which we equated to statements. A statement (sentence) was defined as containing a subject (explicitly or implicitly stated) and a predicate (a verb with or without complements or adverbs). Although simpler structures could also constitute a statement (e.g., “everything is fine”), sentences were the most common structures used for that purpose. Then, we sorted the coding units into categories following the classification used in [11] and presented in Table 2 and 3. Each unit was coded twice, as each has a thematic topic (Table 2) as well as a function (Table 3). After an initial coding iteration, we added the category *Collective past strategy* to the topic categories, which includes statements pointing to a group strategy that was used in past rounds.

**Table 2:** Topic categories; based on [11].

Category Name	Description
1. Game dynamics	
1.1. Collective action	Statements describing the dilemma between individual appropriation and group gains
1.2. Free riding	Statements describing the situation in which an individual can earn high rents at the expense of cooperative behavior of other individuals
1.3. Wrong interpretation	Statements pointing to game dynamics that do not correspond to the workings of the experimental game
2. Past results and actions	
2.1. Group past results	Statements about what the group or individuals other than the speaker did in past rounds of the experiment
2.2. Individual past results	Statements about what the speaker did in past rounds of the experiment
3. Collective strategy	
3.1. General collective strategy	Statements pointing to a general group strategy to be used in subsequent rounds of the experiment
3.2. Specific collective strategy	Statements pointing to a specific group strategy (i.e., including specific numbers) to be used in subsequent rounds of the experiment
3.3. Collective past strategy	Statements pointing to a group strategy that was used in past rounds of the experiment
4. Individualistic strategy	Statements pointing to strategies wherein each participant decides what to do independently from other participants’ decisions
5. Field context	Statements about the real context of life situations in the community and connections to the experiment
6. Game rules	Statements about the rules that specify how decisions and computations are to be made in the experimental game
7. Off topic	Statements that do not fit in any of the preceding categories

**Table 3:** Function categories; based on [11].

Category Name	Description
1. Information	Statements providing descriptions or non-normative opinions, as well as potential acknowledgments following those statements
2. Proposal	Statements suggesting a strategy to be followed in the subsequent rounds of the experiment
3. Evaluation	Statements providing judgments and normative opinions, as well as acknowledgments following those statements
4. Positive Maintenance	Statements showing appreciation, interest, affiliation, or social support for the opinions and/or actions of other group members
5. Negative Maintenance	Statements of disapproval or criticism of the group or other players, as well as expressions of nonconformity, disinterest, displeasure, or frustration with the opinions and/or behaviors of other players
6. Off function	Statements that do not fit in any of the preceding categories

## Hypotheses

Table 4 contains the five hypotheses we created to test for the differences between the two communication treatments. The explanation of each of the hypotheses is found below.

**Table 4:** Hypotheses with corresponding test variables.

Hypotheses	Variables
H1: Contributions for public communication are higher than for club communication	Amount of contributions
H2.1; H2.2: Club communication emerges more frequently than public communication, but full group communication occurs significantly less in club communication meetings	Frequency and size of communication meetings
H3: The content of communication is similar across the two communication treatments	Communication content
H4: The payments for communication decrease over rounds for both communication treatments.	Timing of contributions

*H1*: *Contributions for public communication (PC) are higher than for club communication (CC)*.

While the levels of contributions in the PC treatment are freely chosen (only signal by a group threshold) by each player, CC contributions are fixed; but in both cases voluntary. Few studies have measured the impact of fixed vs. variable contributions to communicate. Available evidence, however, indicates that contributions to public goods are significantly higher in the case of variable contributions than in the case of fix contributions [45–47]. According to [46], this is because exogenous constraints on contributions are less likely to fit the preferences of heterogenous players. If a fixed contribution amount appears to be too high, individuals will prefer not to contribute at all, while a continuous set-up allows for preference-matching contributions. [47] describe the existence of a cooperative outcome in symmetric pure strategies, which only appears when players can freely choose their contributions. This cooperative outcome consists of each player contributing their fair share to reach the provision point, which, for equitable costs, increases payoffs for all players. In the context of fundraising, [48] argue that restricting contribution levels can induce people to contribute more than they otherwise would, but also recognize that allowing flexibility in contributions is more favorable to motivate payments. Additionally, one can argue that voluntary contributions allow for the emergence of leaders, i.e., players who feel more responsible or somehow benefit more from group cooperation than others, and therefore contribute more than others. Assuming that the threshold condition itself does not have a direct influence on the willingness to contribute [49, 50], we expect higher contributions in the PC compared to the CC treatment.

*H2*.*1*: *Club communication (CC) emerges more frequently than public communication (PC)*.

Two important properties of communication are the frequency of communication meetings and the size of the group communicating [10]. Everything else being equal, the CC treatment allows for *any* communication (i.e., among at least two individuals) to happen, compared to the PC treatment where if only two players pay to communicate but they do not reach the threshold, they will not be able to meet. Moreover, the PC treatment bears the incentive for individuals to freeride. Contrary to the CC treatment, individuals in the PC treatment can participate in communication meetings without having contributed. Thus, PC players are confronted with a free rider problem which discourages contributions to communication [51–53]. We therefore expect CC to emerge more frequently than PC.

*H2*.*2*: *Full group communication occurs significantly less in club communication (CC) meetings*.

As posited in the previous hypothesis, we expect that communication emerges more frequently in CC treatment because it allows for communication with less than five participants. Therefore, it ends up being less costly for the group, while in the PC if the target is not reached, the group cannot meet. The PC treatment confronts individuals with a free rider problem, while the CC treatment confronts participants with a coordination problem (i.e., to reach full group communication). In the PC treatment, an individual can provide the threshold to communicate and the entire group will communicate; but in the CC treatment all members of the group need to pay the fee in the same round to come all together as a group. A leader or a few of them could contribute to overcome the free rider problem in the PC treatment, but not the coordination problem in the CC treatment, which leads us to expect full group communication meetings in the CC treatment to occur significantly less.

*H3*: *The content of communication is similar across the two communication treatments*.

More meetings and communication are not necessarily better, for example, in promoting cooperation. This is why scholars who strive to better understand how and why communication affects cooperation have started to question the content of communication interactions [54]. As shown by [11, 40, 55–57], communication interactions in social dilemmas show notable diversity in terms of topics, ranging from discussions about collective action dynamics and the planning of collective strategies, to irrelevant topics. They also show diversity in the functions of communication, including information sharing, evaluations, or reproaches. On the one hand, one might hypothesize that the underlying dynamics of the PC and CC treatments influence the content of communication meetings. On the other hand, the incentives as a group to contribute to communication are equal for both treatments and it is unclear whether communication dynamics end up being different. As we have not found sufficient literature or theory to inform expectations and to formulate concrete predictions, we hypothesize that the communication content will be equal or similar for both treatments.

*H4*: *The payments for communication decrease over rounds for both communication treatments*.

If communicating group members reach a cooperation agreement, they may be less willing to have additional meetings afterwards. This was observed, for example, in the experiments conducted by [20] where groups explicitly coordinated on not to pay for additional communication meetings once a cooperation agreement was made. On the other hand, *cheap talk* without institutions that enforce agreements does not guarantee compliance with a proposed group strategy, particularly in the advent of changes in the environment or deviations from expected behavior [23]. This can be frustrating for participants and in turn make communication payments more appealing than otherwise. Despite this, we expect payments for communication to be made in early rounds, and to decline continuously regardless of cooperative success.

## Results

We started the analysis by comparing group extraction differences before and after communication for each group. Table 5 includes mean extraction levels per group for rounds without (rounds 1–10) and with (rounds 11–20) communication, and extraction levels for rounds 10 and 11 (right before and after the start of the communication treatment). Testing whether the two treatments are comparable before communication started, we conducted independent 2-group t-tests, which yield that the means of the average group extraction levels and the group extraction levels in Round 10 are not significantly different (p = 0,49 and p = 0,76, respectively). It also shows how many times each group or subset of a group has met to communicate throughout rounds 11–20, regardless of the number of people that were in that meeting.

**Table 5:** Group extraction levels before and during the communication treatments.

		Average group extraction levels			t-test	Mann-Whitney U-test	Group extraction levels		Number of meetings
	Group	Rounds 1–10		Rounds 11–20	p-value	p-value	Round 10	Round 11	Rounds 11–20
**CC**	1		35.5 (7.9)	23.4 (3.9)	**0.003**	**0.002**	39	23 [4]	2
	2		42.5 (6.9)	29.6 (8.5)	**0.005**	**0.005**	36	34 [4]	4
	3		31.8 (9.3)	32.6 (8.9)	0.870	0.999	43	37 [3]	7
	4		34.9 (6.1)	23.4 (5.6)	**0.005**	**0.003**	24	27 [0]	0
	5		25.4 (5.2)	20.3 (4.8)	**0.054**	**0.075**	37	24 [0]	0
	6		23.3 (6.8)	25.4 (6.0)	0.498	0.543	14	28 [0]	0
	7		13.3 (3.7)	11 (3.1)	0.242	0.254	11	9 [0]	0
	8		32.7 (11.2)	35.4 (5.3)	0.564	0.520	49	41 [0]	1
	9		36.9 (7.8)	31.3 (12.3)	0.261	0.162	24	15 [3]	2
	10		40.9 (6.9)	42 (6.9)	0.766	0.677	49	55 [2]	1
**PC**	11		23.6 (4.0)	7.1 (5.2)	**0.000**	**0.000**	21	4 [5]	2
	12		16.5 (5.5)	16.4 (2.0)	0.956	0.879	26	14 [0]	1
	13		45.8 (7.5)	55.1 (5.2)	**0.004**	**0.015**	59	55 [0]	0
	14		42.6 (5.5)	28.9 (5.0)	**0.001**	**0.001**	49	41 [0]	1
	15		15.7 (4.8)	6.8 (1.5)	**0.001**	**0.000**	23	9 [5]	1
	16		36.7 (10.6)	24.7 (8.1)	**0.027**	**0.021**	40	36 [0]	0
	17		38.2 (7.1)	48.3 (9.1)	**0.019**	**0.028**	35	33 [0]	0
	18		14.3 (5.4)	11.5 (5.3)	0.317	0.240	5	22 [0]	1
	19		32 (11.7)	31.3 (9.7)	0.886	0.970	19	24 [0]	2
	20		18.7 (7.6)	15.6 (7.4)	0.491	0.470	29	13 [0]	0

Standard deviations in parenthesis. Square brackets in Round 11 column indicate number of people communicating in that round. The tests for normality resulted in mixed results: Shapiro-Wilk for CC series: z = 1.888, p-value = 0.117; Shapiro-Wilk for PC series: z = 4.399, p-value = 0.000. Here we present both pairwise t-test and Mann-Whitney U-test for group extraction levels. Significant p-values (at least at the 10% level) in bold.

As shown in Table 5, from round 10 to 11, six groups in the CC treatment and eight in the PC treatment decreased their extraction levels, which shows that even if the group did not pay to communicate (only two groups met in that round in the PC treatment, and five in the CC treatment, but none with the full group) the announcement that communication was possible decreased, at least initially, some of the group extraction behavior. Then, when comparing the whole 10 rounds, we observe that in both treatments only four groups significantly decreased their extraction levels, whereas two groups in the PC treatment increased their extraction levels. These findings support only partially previous findings about the positive effects of communication on harvest behavior and further motivate our interest in better understanding the provision and experience of communication across the treatments. In the analysis that follows, we concentrate on those aspects with a focus on communication payments, number of meetings, and the information shared in those meetings.

**Fig 1:**
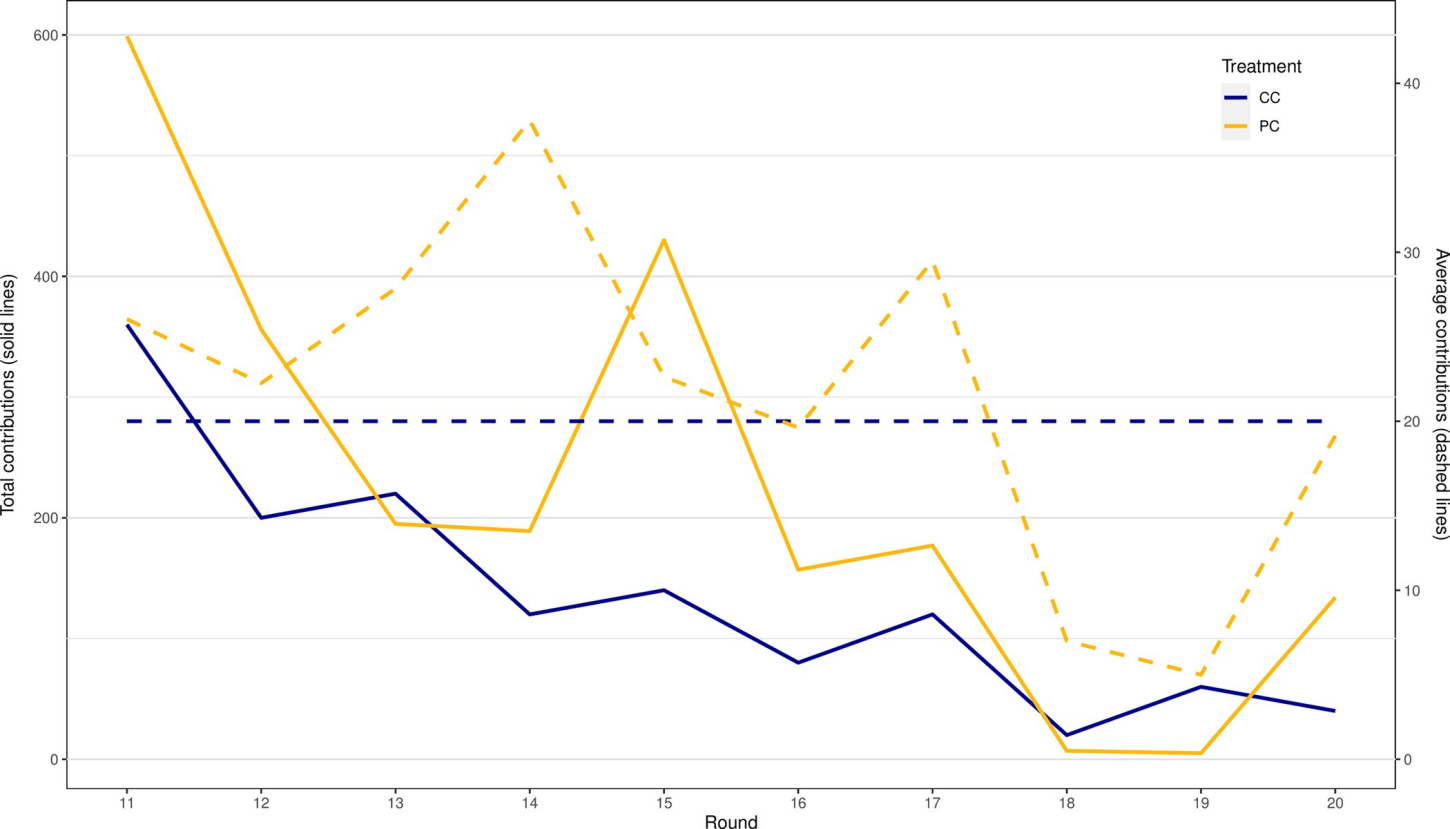
Evolution of contributions per treatment.

First, we compared communication contributions and meetings across the two treatments (Table 6). In the PC treatment, players paid a total of 2,267 ECUs for the possibility to communicate, resulting in 8 communication meetings. In the CC treatment, participants paid a total sum of 1,360 ECUs, and met in 17 occasions (this includes all payments to communicate, regardless of whether a communication meeting took place or not). A first important result to show case here is the fact that participants did not make much use of the mechanism that allowed them to pay to communicate among themselves. This is an important result because, as we mentioned earlier, communication had shown to be effective, and even more so in contexts similar to the one, but when communication was free [58]. The results presented in Table 6 directly align with our hypotheses H1 and H2.1. Fig 1 shows the development of contributions for each mechanism per round. Averages are calculated based on non-zero contributions. In the case of total contributions, more ECUs were spent in the PC treatment than in the CC treatment, with an observable decline over rounds. The results further show that communication groups in the CC treatment were always smaller than 5 individuals, i.e., full group communication was never reached. This result supports our hypothesis H2.2.

**Table 6:** Selected attributes of both communication treatments.

	Public Communication	Club Communication
Groups with communication	6	6
Σ of people communicating	40	43
Rounds with communication	8	17
Number of people communicating per round	5	2.5 (range: 2–4)
Σ payments	2267	1360

**Fig 2:**
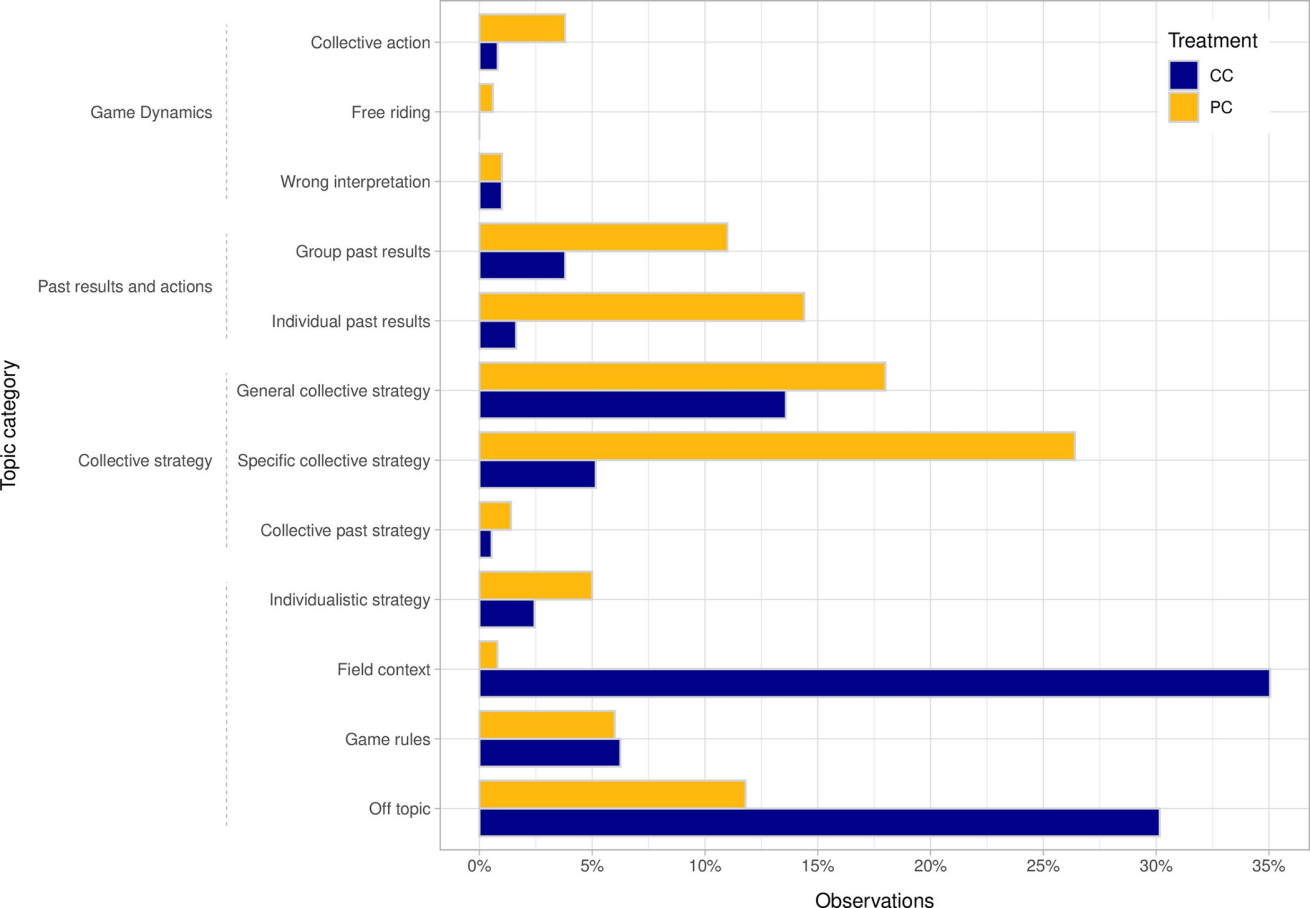
Relative distribution of coding units by topic categories and across communication treatments.

**Fig 3:**
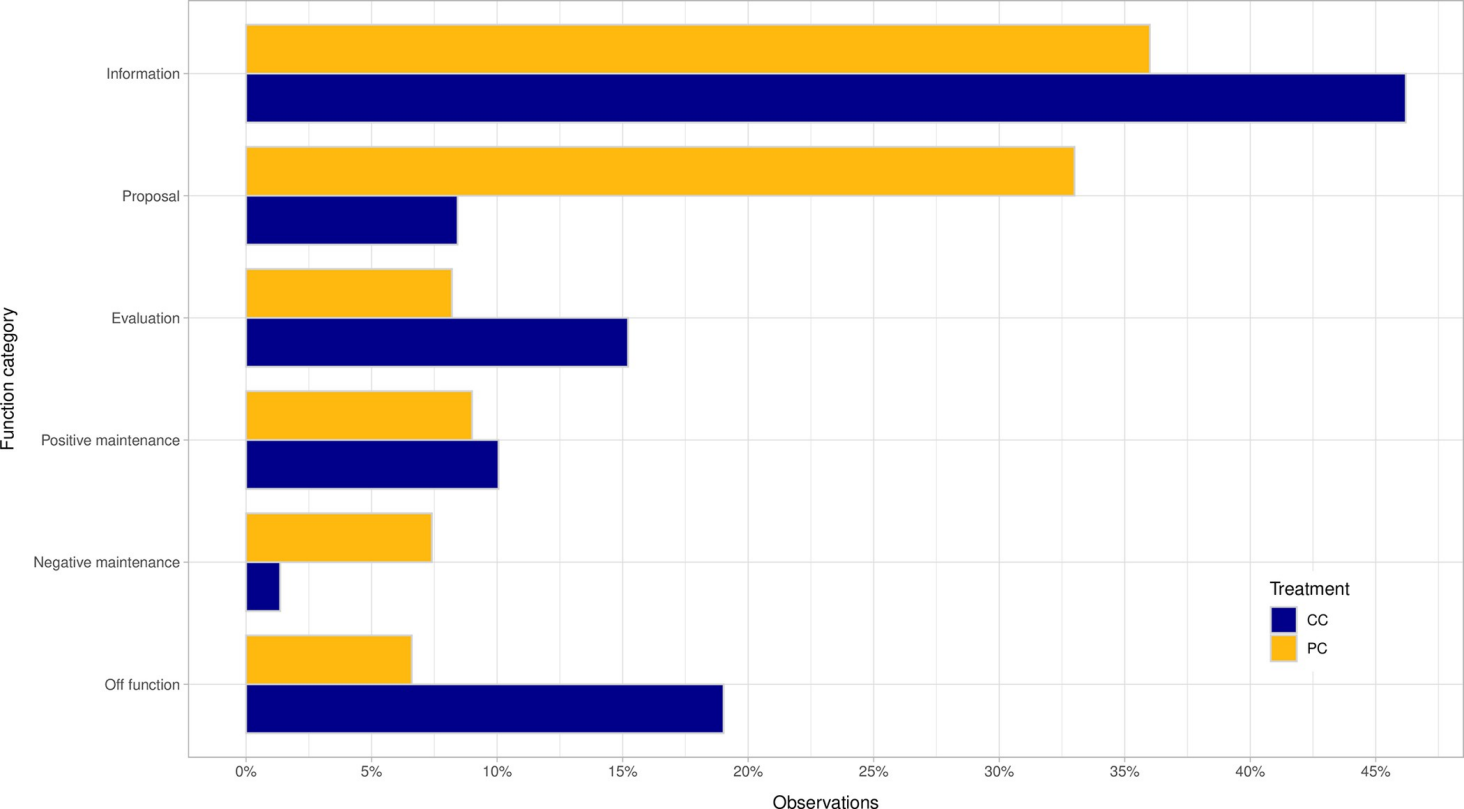
Relative distribution of coding units by function categories and across communication treatments.

Next, we analyzed the communication content. The coding resulted in 501 coded units in the PC treatment and 368 coded units in the CC treatment. Throughout the coding process, 17 units in the CC rounds and 62 units in the PC rounds were coded as unclear due to bad quality of the recordings or unidentifiable statements, and were therefore omitted from the analysis. The coding results per category are shown in terms of percentages in Figs 2 and 3 for the topic and function categories respectively. Looking at the results, we find substantial content differences across the two treatments, rejecting hypothesis H3. Looking first at the game dynamics category, which accounts for statements acknowledging the dilemma situation, the incentive of free riding, or a wrong interpretation of the game, we see comparatively low numbers for both treatments. Overall players in the PC treatment dedicated most of their time to discuss collective strategies and past results and actions. Recurrent collective strategies were pointing to the need to reduce extraction levels to a certain amount, although others also proposed repeated communication meetings. Alternatively, players in the CC treatment tended to contextualize their commentaries about the game (*field context*) or not talk about the game at all (*off topic*). Talks about strategy and past results and actions on the other hand were relatively lower for CC groups.

In the function category, informative statements were the most frequent in both treatments. PC is further characterized by a high number of statements corresponding to proposals. Also, the CC treatment has noticeably fewer proposal statements but more off function talks than the PC treatment; and, while both treatments produced a similar number of statements about positive group maintenance, negative group maintenance seems to be more pronounced in the PC treatment.

Basic statistics of the communication content are displayed in Table 7. In order to check for statistically significant differences across the treatments, we calculated the percentage of statements per round (i.e., number of statements for a category over total number of statements in a round) and conducted a Mann-Whitney U-test. As shown, differences across the two treatments are statistically significant for 12 out of 18 categories. *Group past results* and *Specific collective strategy* (among the topical categories), and *Proposal* and *Negative Maintenance* (among the function categories) are significantly more frequent in the PC treatment. The *off function* and *Field context* categories are, alternatively, significantly more frequent in the CC treatment.

**Table 7:** Statistical analysis of communication statements across treatments.

	% statements per treatment^1^		% statements per round^2^				
			Mean		Median		
	Public Comm.	Club Comm.	Public Comm.	Club Comm.	Public Comm.	Club Comm.	Mann-Whitney U-test (p-value)
**Function categories**							
*Information*	36%	46%	35%	42%	34%	45%	0.398
*Proposal*	33%	8%	35%	7%	27%	0%	**0.001**
*Evaluation*	8%	15%	8%	12%	5%	6%	0.837
*Positive Maintenance*	9%	10%	9%	9%	8%	6%	0.748
*Negative Maintenance*	7%	1%	6%	1%	6%	0%	**0.01**
*Off function*	7%	19%	6%	30%	3%	17%	**0.02**
**Topic categories**							
*Collective Action*	4%	1%	4%	3%	2%	0%	**0.098**
*Free Riding*	1%	0%	<1%	0%	0%	0%	0.17
*Wrong interpretation*	1%	1%	<1%	<1%	0%	0%	**0.075**
*Group past results*	11%	4%	10%	4%	9%	0%	**0.003**
*Individual past results*	14%	2%	14%	1%	14%	0%	**0**
*General collective strategy*	18%	14%	22%	11%	17%	0%	**0.024**
*Specific collective strategy*	26%	5%	26%	4%	22%	0%	**0.001**
*Past collective strategy*	1%	1%	1%	<1%	0%	0%	0.113
*Individualistic strategy*	5%	2%	5%	3%	3%	0%	**0.061**
*Field context*	1%	35%	<1%	30%	0%	9%	**0.016**
*Game rules*	6%	6%	6%	5%	5%	0%	0.279
*Off topic*	12%	30%	11%	37%	8%	33%	**0.007**

Significant p-values (at least at the 10% level) in bold. The Shapiro-Wilk normality test for the CC series (w = 0.974, p-value = 0.886) and for the PC series (w = 0.873, p-value = 0.162) resulted in rejection of null hypothesis.

^1^ Number of statements for category/total number of statements in treatment

^2^ Average of number of statements for category/total number of statements in a round

Tables 8 and 9 show the percentage of each intersection of topic and function categories for each communication treatment. In the PC treatment, intersections with the highest percentage are *Proposal/Specific collective strategy* (0.33), *Information/Individual past results* (0.27), and *Off function/Off topic* (0.34). This number means that, e.g., of all *Proposal* and *Specific collective strategy* statements, 33% fall into the intersection of those two categories. Thus, we see that when proposals are made, they are most frequently directed to a specific strategy to follow. Statements about past results, in contrast, recurrently have an informative function.

**Table 8:** Ratios of intersections of topic and function categories in the PC treatment.

	*Evaluation*	*Information*	*Negative Maintenance*	*Positive Maintenance*	*Proposal*	*Off function*
*Collective Action*	0.12	0.04	0.00	0.06	0.00	0.00
*Free Riding*	0.00	0.01	0.00	0.02	0.00	0.00
*Wrong interpretation*	0.02	0.01	0.00	0.04	0.00	0.00
*Game rules*	0.01	0.13	0.00	0.03	0.00	0.00
*Off topic*	0.07	0.01	0.06	0.13	0.00	**0.34**
*Group past results*	0.08	0.13	0.14	0.04	0.00	0.00
*Individual past results*	0.01	**0.27**	0.03	0.01	0.00	0.00
*Field context*	0.00	0.02	0.02	0.00	0.00	0.00
*General collective strategy*	0.07	0.04	0.04	0.07	0.22	0.00
*Past collective strategy*	0.04	0.02	0.05	0.00	0.00	0.00
*Specific collective strategy*	0.01	0.04	0.04	0.05	**0.33**	0.01
*Individualistic strategy*	0.05	0.05	0.00	0.00	0.06	0.00

**Table 9:** Ratios of intersections of topic and function categories in the CC treatment.

	*Evaluation*	*Information*	*Negative Maintenance*	*Positive Maintenance*	*Proposal*	*Off function*
*Collective Action*	0.00	0.02	0.00	0.00	0.00	0.00
*Free Riding*	0.00	0.00	0.00	0.00	0.00	0.00
*Wrong interpretation*	0.00	0.00	0.00	0.03	0.03	0.00
*Game rules*	0.01	0.11	0.00	0.02	0.00	0.00
*Off topic*	0.08	0.09	0.00	0.08	0.00	**0.34**
*Group past results*	0.01	0.06	0.11	0.00	0.00	0.00
*Individual past results*	0.02	0.03	0.00	0.00	0.00	0.00
*Field context*	0.14	**0.28**	0.00	0.08	0.01	0.04
*General collective strategy*	0.08	0.05	0.05	0.08	**0.23**	0.01
*Past collective strategy*	0.02	0.01	0.00	0.00	0.00	0.00
*Specific collective strategy*	0.04	0.02	0.00	0.04	0.20	0.00
*Individualistic strategy*	0.03	0.03	0.00	0.02	0.00	0.00

The highest percentages of intersections in the CC treatment are *Information/Field context* (0.28), *Proposal/General collective strategy* (0.23) and *Off function/Off topic* (0.34). The intersections with the highest proportions are underlined and bold in the tables. Players in the CC treatment proposed general strategies proportionally more than specific strategies, and statements about the field context were the most frequently of informative nature. In both treatments, off topic-talk was perceived to have no identifiable function.

Finally, we analyzed whether payments for communication follow a specific trend. As mentioned in the previous section, paying for communication one round after the other does not make sense from a rational perspective when communication is costly and the communication process was effective to decide future strategies to start with. Therefore, we expected payments for communication to happen in the first rounds where communication was possible and to decline throughout the experiment. We see this to hold true for the CC treatment. In the first round of communication, the sum of contributions reached its peak at 360. In the subsequent rounds, we see a clear decline in the number of payments for communication. The case for the PC treatment is less clear. Although we see the largest sum of contributions at 599, also in the first round, there is another noticeable peak in round 15 (430). After almost zero contributions in rounds 18 and 19, a visible amount was paid in the last round. The actual development can be seen in Fig 1.

## Discussion

### Discussion of hypotheses

As shown, participants were more willing to contribute to a communication meeting in the PC treatment, which supports our first hypothesis (H1). Contributions were 66% higher in the PC treatment compared to the CC treatment. Not limiting contributions to an all-or-nothing decision gives players the possibility to differentiate and express their individually perceived benefits and therefore their willingness to contribute [47]. In fact, we did observe participants contributing more than the equal share of 20ECUs in the PC treatment, which supports our idea that group leaders emerge and “take matters into their own hands” to make discussion meetings possible. This aligns with findings on leader emergence in public good provision dilemmas [59].

The result that communication rounds with any number of participants emerge more frequently in the CC treatment aligns with hypothesis H2.1. One favorable aspect of repeated meetings is that recurrent interactions increase trust and build reciprocal relationships among participants, which in turn could lead to higher levels of performance [6]. Although the content of communication would be expected to affect whether such relationships emerge, the mere fact of meeting repeatedly may make already a difference. Learning in participatory governance, for instance, has been found to be strengthened by repeated, goal-oriented communication [60].

Hypothesis H2.2 focused on the number of people communicating. As anticipated, the results yield that in the CC treatment the average number of people communicating were 2.5, whereas because of the nature of the PC treatment (meeting all or not meeting) when these groups met they all had 5 people in the discussion. In addition to the coordination problem of contributing at the same time to reach full group communication, the CC treatment permits non-cooperative players to strategically abstain from meetings to avoid shaming by other players for non-cooperation [36], and potentially benefit from cooperative behavior of communicating group members. Players that decided not to pay for communication out of their unwillingness to lose ECUs gave up on the opportunity to improve group and individual payoffs via cooperation.

Hypothesis H3 addressed communication content and allowed us to qualify our previous findings. Based on our data, we can reject the hypothesis of no differences across the treatments. Other studies have also found differences in communication content across treatments but failed to systematically analyze them [42, 43, 61, 62]. In our analysis, proposals suggesting a general or specific game strategy were significantly more frequent in the PC treatment than in the CC treatment. On the other hand, off topic comments were significantly higher in the CC treatment. This result can be associated to the recurrent absence of some individuals in the communication meetings of the CC treatment. As a result, those who communicate shall be less encouraged to talk about strategies and be more prone for conversations that are not goal oriented (e.g., talk *off topic*). Also, negative maintenance statements were more frequent in the PC treatment than in the CC treatment. This can be associated with the previous result about proposals (reproaches tend to be associated to the failure to comply with proposals and agreements). This finding can also be associated with the number of people communicating. Assuming that trust and social cohesion are more pronounced in smaller groups [57], one could expect social norms (and therefore reproaches) playing a stronger role than otherwise. That said, as the number of individuals absent from communication meetings increases, the sense of effectiveness (e.g., of reproaches) among those communicating shall decrease.

Finally, we turn the focus on the timing of contributions. We observe that the groups in both treatments behaved similarly in this aspect. The number of communication contributions in the CC treatment follows a declining path, reaching its highest number in the first round and steadily declining towards zero with small irregularities, while communication contributions in the PC treatment follow a less regular declining path. This supports our hypothesis H4 which posited a declining interest in repeated costly meetings. In addition to the points made in the hypothesis section, one can infer that communication in the PC treatment was used not just for informative purposes but also as an instrument to address a lack of compliance with previous agreements [21]. This is supported by the rather spread-out distribution of meetings in the PC treatment. It also makes sense considering that the PC treatment guaranteed full group communication among all participants each time. Meetings including the exact same sets of people in the CC treatment were indeed few, even among subsets of participants.

### Policy implications

As argued in the introduction, we understand the two communication treatments as proxies for centralized (PC) and networked (CC) participatory processes. The discovered disparities between the two modes of communication prove that the distinction is relevant and gives rise to several policy implications.

Our first finding indicates that communication meetings arise more frequently when they are allowed to emerge as (incomplete) networks of those stakeholders who are willing to disburse some cost. As continuous participation has been found to improve the outcome of participatory governance processes [60, 63], it may be that in some situations it is preferable to ensure the frequency of meetings than their inclusiveness, particularly if the meetings would be attended by most interested stakeholders or cooperation leaders. A considerable drawback of this strategy, however, is that it can leave a fair number of less interested but still decisive stakeholders outside of decision making, or allow individualistic stakeholders to strategically abstain from meetings that they might eventually benefit from without having contributed [36].

Communication content, on the other hand, seems to be more constructive content-wise the more “complete” the group of stakeholders is, i.e., the fewer members are missing in a communication meeting. If a meeting does not reach a sufficiently large number of resource users, it is much harder to engage in conversations about collective issues like those related to the game. In this context, including as many stakeholders as possible in the process to ensure goal-oriented discussion and exchange could be a potential strategy to help overcome resource dilemmas [64]. This however would need to be backed up by a thorough analysis of extraction behavior. This can be seen as an important advantage of centralized participatory processes, and might explain why this design is applied predominantly in practice [24, 25].

Both types of processes bring about their (dis-)advantages, and policy makers might consider creating participatory platforms that consider the favorable characteristics of both centralized and networked participatory processes. Further research shall try to understand to what extent this can be achieved. As a start we would posit that different modes of communication shall be more suitable depending on the needs of communication. Whenever the goal is to make proposals and reach commitment around particular courses of collective action, public communication may be more appropriate; however, whenever the goal is to maintain operations on an agenda over specific themes ongoing and/or commitment for action is less necessary, then club communication could work better.

## Conclusion

Our goal was to uncover meaningful differences between two mechanisms, *public* and *club communication*, providing communication as a costly good in a CPR experiment. For this purpose, we analyzed data from a lab-in-field experiment regarding number of communication contributions made, the frequency, size, and inclusiveness of the communication meetings, and the communication content.

We found that the two communication mechanisms differ in all analyzed aspects. Contributions to communication for PC were considerably higher than for CC. Furthermore, communication groups in the CC treatment met more frequently, but never as a full group including all members. We also found significant differences across the two treatments in terms of communication content. While full communication groups under the PC treatment generally had more goal-oriented discussions on collective strategies and past actions and results, groups under the CC treatment spent most of their time speaking about the general context of the experiments and unrelated themes. Finally, we found that also the timing of meetings varied. While CC group meetings took place in the early rounds with a declining trend over time, PC groups met in a more irregular pattern.

Structuring communication as a club good confronts group members with an additional coordination problem compared to the public good structure, namely the challenge to reach full inclusiveness of a communication meeting. We presume that inclusiveness plays a major role in the discrepancies on the characteristics of communication meetings, and therefore we expect it to have a significant influence on cooperation and game outcomes. This might be considered relevant for planning future policies supporting stakeholder participation via providing communication platforms. Also, in this study we modelled the distinction between public and club communication by combining “flexible vs. fixed contribution” and “threshold vs. continuous” features; however, other ways to model said distinction are possible. Similarly, further research may explore the separate impact of each of the mentioned features on communication contributions and content. These research inroads have great potential vis-à-vis a better understanding of communication dynamics in CPR contexts, a topic with particular policy-importance given the increasing uncertainty around climate change, environmental degradation, and natural resource management at large.

## Supporting information

S1 File Game instructions. (DOCX)
