# Supplementary material for: Analyzing group communication dynamics and content in a common-pool resource experiment
Source: PLoS One. 2023 May 2;18(5):e0283196. doi: 10.1371/journal.pone.0283196 (PMC10153700; doi:10.1371/journal.pone.0283196)
Supplement: S1 File — (DOCX) [file pone.0283196.s001.docx]

**SUPPORTING INFORMATION**

Analyzing group communication dynamics and content in a common-pool resource experiment

**Translation of Experiment Explanation and Instructions**

**Note:** *The experiment included further features that were not analyzed in this study. This is seen, for instance, when the instructions explain 30 rounds of gameplay. Our study focuses only on the first 20 rounds of the experiment. All parts of the full instructions referring to features outside of the scope of this study are omitted. Original language Spanish. Translated to English using deepl.com.*

**Introduction**

Before we begin today’s exercise, we want to thank everybody for coming and participating. The funding for these exercises comes from international foundations. This exercise recreates a situation in which a group of people or a community must decide about the use of water in a watershed. All the earnings you will make and the decisions you will provide us are strictly confidential. We will not reveal your final earnings to any member of the community or anyone else.

Today’s exercise may be different from the exercise other members of your community have already participated in. For that reason, we ask you to make your decisions based of the instructions we will give you and not based on any comments you might have heard. Please pay careful attention to these instructions, which will allow you to make good decisions. At the end of the exercise, you will receive your earnings in cash based on your decisions and the decisions of the others in your group.

You can leave the exercise at any time. However, if you decide to leave before the exercise ends, you will not receive the earnings that you would have accumulated to that point.

Please remain seated and do not talk to other players. If you have any questions please raise your hand and we will answer them.

**Instructions**

In today’s exercise you will have to decide how you allocate your working time and, according to this, you will receive your earnings. The exercise has a total of 30 rounds. At the end of the exercise, while you answer a survey, we will calculate your earnings.

You have been selected to participate in a group of 5 people. Today there will be _____ groups participating at the same time. However, each group is independent from other groups. Decisions from the other groups do not affect the decisions of your own group. Each group will be differentiated by the color of the sheet used during the exercise.

In this exercise you will earn money depending upon your decisions and the decisions of the other members of your group. The reason why we use money during the exercise is to recreate real-life situations where your economic decisions have consequences for your pocket. We do not consider the money you earn as a payment for your participation in this study or the only reason to participate.

During the exercise, all of your earnings will be calculated in tokens. For each token you earn you will receive 4 pesos:

1 token = 4 pesos

After finishing the 30 rounds we will sum the total number of tokens you have earned and convert them to pesos. So, if after finishing the 30 rounds you have earned 3,000 tokens, we will give you 12,000 pesos.

The number of pesos that you will receive will be rounded to the nearest 500 pesos.

Your earnings will be paid to you in cash in private.

**How decisions should be made**

Your group has a watershed. At the beginning of each round your group’s watershed water has a value of 675 tokens. The decisions that you and each of the other four members of your group make about how to allocate your working time will affect the watershed.

At each round you will have to decide how to allocate 20 working days. Each day, you can choose between one of these two activities:

- - - Extract water from the watershed to be used in a productive activity, or
    - Work as an employee with a fixed salary

Take into account that each day you can choose only one of these two activities, but you can reallocate your 20 days at each round with different combinations of these activities.

For example:

In one round, you can decide to extract water from the watershed one day and work as an employee for 19 days.

Or you can decide to extract water 17 days and work as an employee 3 days.

Or you can choose any other combination that adds to 20 days.

You can also repeat a combination as many times as you want.

At each round, the days that you extract water from the watershed plus the days that you work as an employee must add to 20 days. And you must make this decision for 30 rounds.

To understand how to obtain your earnings based on these decisions, let’s look at the following:

For each day you decide to work as an employee you will earn 1 token. Since you did not extract water from the watershed, you will not affect the watershed.

For each day you decide to extract water from the watershed you will earn 2 tokens. Since you extracted water from the watershed, the value of the water in the watershed is reduced by 3 tokens.

**1 day working as an employee = 1 token for you**

**1 day working as an employee = 0 tokens less in the watershed**

**1 day extracting water from the watershed = 2 tokens for you**

**1 day extracting water from the watershed = 3 tokens less in the watershed**

**Your group’s watershed water has a value of 675 tokens**

For one day of extracting water from the watershed, the watershed loses 3 tokens and you earn 2 tokens. There is one token missing from your earnings because that is the value of the water lost during transportation of the water.

Do you have any question at this point?

At the end of each round, the water that is left in the watershed has a value that benefits you and the others in your group in the same way. The value of the water that is left in the watershed is divided evenly among the five players of your group. Each player will receive the same number of tokens independent of his/her decisions.

The more the group extracts water from the watershed, the lower the value will be of the water left in the watershed at the end of the round. Therefore, fewer tokens will be divided among the group players.

Let’s see some examples.

*Example 1.*

*Each player works 10 days*

*Each player extracts water 10 days*

*The total number of days the group extracts water from the watershed is 50 days*

*The group extracted water for a total value of 150 tokens*

*The water remaining in the watershed has a value of 675 - 150 = 525*

*The value of the water left in the watershed is divided by 5 = Each of you will earn 105 tokens from the water left in the watershed*

*Example 2.Each player works 8 days*

*Each player extracts water 12 days*

*The total number of days the group extracts water from the watershed is 60 days*

*The group extracted water for a total value of 180 tokens*

*The water remaining in the watershed has a value of 675 - 180 = 495*

*The value of the water left in the watershed is divided by 5 = Each of you will earn 99 tokens from the water left in the watershed*

*Example 3.Each player works 5 days*

*Each player extracts water 15 days*

*The total number of days the group extracts water from the watershed is 75 days*

*The group extracted water for a total value of 225 tokens*

*The water remaining in the watershed has a value of 675 - 225 = 450*

*The value of the water left in the watershed is divided by 5 = Each of you will earn 90 tokens from the water left in the watershed*

Do you have any questions at this point?

Let’s remember the way you calculate your earnings based on your decisions.

You decide, from the 20 working days of one round, how many days you will work as an employee, and how many days you will extract water from the watershed for a productive activity:

1. For each day that you work as an employee you will earn 1 token and the watershed will not be affected.
2. For each day that you extract water from the watershed you will earn 2 tokens and the value of the water in the watershed will be reduced by 3 tokens, Remember: 1 token is lost during transportation.

At the beginning of a new round, your group’s watershed will again have a value of 675 tokens and you will again have 20 working days to allocate between working as an employee and extracting water from the watershed.

Remember that at the end of the exercise we will add the tokens you earned during the 30 rounds and convert them to pesos that you will receive in cash.

**Decision card**

| **Decision card** | |
| --- | --- |
| Player number: | 1 |
| Round number: |  |
| Days that I worked as an employee |  |
| Days that I extracted water from the watershed |  |

Now I will explain the way you will inform us about your decisions at each round. We will give you “decision cards,” which are those small pieces of paper. As you can see, each decision card has a player number. That number will be your identification number during the exercise. In the next row, you must write the round number that the monitor will announce.

In the next two rows, you will write the number of days that you decide to work as an employee and the number of days that you decide to extract water from the watershed.

Remember, at each round you will have to enter numbers for the following:

- “Days that I worked as an employee”: This means how many days you decide to work as an employee (a number between 0 and 20).
- “Days that I extracted water from the watershed”: This means how many days you decide to extract water from the watershed (a number between 0 and 20).

The sum of “Days that I worked as an employee” and “Days that I extracted water from the watershed” must equal 20.

When all your group members have made their decisions and written them on their decision cards, we will collect them, calculate the amount of water extracted in tokens from the watershed and finally announce the result in public.

The total amount of water left in the watershed will be publicly reported and we will calculate the number of tokens that each player will receive from that amount.

Please remember that your decisions are private and that you cannot show them to the other members of your group.

Do you have any questions at this point?

**Calculation sheet**

You will receive a calculation sheet to keep a record of your decisions and the number of tokens you earned. Please write down your identification number on the sheet. Your identification number is the same number shown on your decision cards.

Let’s see how to use the calculation sheet by looking at some examples.

**Practice rounds**

Before we start the exercise we will do some practice rounds. The decisions you make in these practice rounds will not affect your earnings today.

In order to properly understand how the exercise works, we will do some practice rounds together using the same number of days working as an employee and the same number of days extracting water from the watershed. Remember that, later, each of you will have to make your own decisions knowing that “Days that I worked as an employee” + “Days that I extracted water from the watershed” must be equal 20.

*Example with 5 days working as an employee and 15 days extracting water from the watershed.*

*Example with 15 days working as an employee and 5 days extracting water from the watershed.*

*Example with 10 days working as an employee and 10 days extracting water from the watershed.*

**Treatment Communication (fee)**

Let's start the second part of the exercise. From now on, at the beginning of each round the basin of your group has water worth 450 tokens. That is, your group's basin no longer has water worth 675 tokens but has water worth 450 tokens.

Sometimes communication can be helpful in making decisions. In this second part you will have the opportunity to meet and talk with the other players in your group for 3 minutes. These meetings have a cost. In order to meet, you will have to pay 20 tokens. Those who choose to pay will be able to meet with each other privately and those who do not pay will not be able to meet and listen to each other's conversation. [EXPLAIN WHERE THOSE WHO MEET OR DO NOT MEET GO]. In the event that only one player pays the same, the 20 tokens paid for meeting will be subtracted from his winnings. So, at the beginning of each round, before making your decisions on how to use your 20 working days, you will have to decide whether or not to pay to be able to meet with the other players in your group.

Now let's see how you will make your decisions.

First you will use a meeting card.

| **MEETING CARD** | | |
| --- | --- | --- |
| Player number: |  | |
| Round number: |  | |
| Do I want to pay for a meeting? | **YES** | **NO** |

On this card you must mark "YES" if you wish to pay the 20 tokens to be able to meet with other players in your group, and "NO" if you do not wish to pay to be able to meet. This decision should be recorded on your score sheet.

***Accounts sheet***

For this purpose, we have added a new column in the Accounts Sheet. This new column, column "A", is called "Tokens I paid to meet". If you wrote "YES" on the reunion card, you should write the 20 tokens that you paid to reunite in this column (SHOW COLUMN). If you wrote "NO" on the meeting card, you must write 0 tokens in this column (SHOW COLUMN).

After each player writes whether he/she wants to pay for meeting or not on the meeting card and in column A of the score sheet, the monitor will announce which players will be allowed to meet.

In case there is a meeting, once the 3 minutes meeting time is over, all players will return to their places and make their decisions on how to use their 20 working days exactly as they have done so far on the decision card and on the account sheet.

All other conditions are the same. Remember:

**1 day working as an employee = 1 token for you**

**1 day working as an employee = 0 tokens less in the watershed**

**1 day extracting water from the watershed = 2 tokens for you**

**1 day extracting water from the watershed = 3 tokens less in the watershed**

**Your group’s watershed water has a value of 450 tokens**

The value of the water remaining in the basin is divided equally among the 5 players in the group. Each player will receive the same number of tokens regardless of their decisions.

Now we will play for another 10 rounds.

**Treatment Communication (provision point)**

Let's start the second part of the exercise. From now on, at the beginning of each round the basin of your group has water worth 450 tokens. That is, your group's basin no longer has water worth 675 tokens but has water worth 450 tokens.

Sometimes communication can be helpful in making decisions. In this second part, you will have the opportunity to meet to talk with the other players in your group for 3 minutes. These meetings have a cost. The group will be able to meet if together they reach 100 tokens. Everyone can contribute as much as they wish. So, at the beginning of each round, each player must decide how many tokens he or she wants to pay for the group to meet. If your group reaches 100 tokens all players will be able to meet. If your group does not reach 100 tokens the group will not be able to meet. The tokens you pay to meet will be subtracted from your winnings whether the group has met or not.

Now let's see how you will make your decisions.

First you will use a group meeting card.

| **GROUP MEETING CARD** | | |
| --- | --- | --- |
| Player number: |  | |
| Round number: |  | |
| Do I want to pay for a group meeting? | **YES** | **NO** |
| How much I want to pay for the group to meet: |  | |

On this card you should mark "YES" if you wish to pay for the group to meet and write the number of tokens you wish to pay, and "NO" if you do not wish to pay for the group to meet. This decision you will need to record on your account sheet.

**Account sheet:**

To do this, we have added a new column in the Account Sheet. This new column, column "A", is called "Tokens I pay to meet". If you wrote "YES" on the reunion card, you should write the tokens you paid to reunite in this column (SHOW COLUMN). If you wrote "NO" on the meeting card, you must write 0 tokens in this column (SHOW COLUMN).

After each player has written whether he/she wants to pay for the group to meet or not on the meeting card and in column A of the account sheet, the monitor will announce whether the group has reached 100 tokens and can meet or not.

In case there is a meeting, once the 3 minute meeting is over, all players will return to their places and make their decisions on how to use their 20 working days exactly as they have done so far on the decision card and on the tally sheet.

All other conditions are the same. Remember:

**1 day working as an employee = 1 token for you**

**1 day working as an employee = 0 tokens less in the watershed**

**1 day extracting water from the watershed = 2 tokens for you**

**1 day extracting water from the watershed = 3 tokens less in the watershed**

**Your group’s watershed water has a value of 450 tokens**

**In order to meet, your group will have to pay 100 tokens**

The value of the water remaining in the basin is divided equally among the 5 players in the group. Each player will receive the same number of tokens regardless of their decisions.

Now we will play for another 10 rounds.
